# Safety and efficacy of strictureplasty for primary proximal duodenal Crohn’s disease: a retrospective single-center case series

**DOI:** 10.1007/s13304-026-02711-w

**Published:** 2026-07-02

**Authors:** Weilin Qi, Wei Liu, Xiaolong Ge, Wei Zhou

**Affiliations:** Department of General Surgery, School of Medicine, Sir Run Run Shaw Hospital, Zhejiang University, 3 East Qingchun Road, Hangzhou, 310016 China

**Keywords:** Crohn’s disease, Duodenal Crohn’s disease, Strictureplasty, Bowel-sparing surgery, Laparoscopic-assisted surgery

## Abstract

Primary proximal duodenal Crohn’s disease (CD) is a rare entity that can lead to fibrotic strictures and gastric outlet obstruction. Surgical intervention is often required in refractory cases, yet the optimal approach remains debated. This study aimed to evaluate the safety and efficacy of strictureplasty in this context. We retrospectively reviewed patients with primary proximal duodenal stricturing CD who underwent strictureplasty at Sir Run Run Shaw Hospital, Zhejiang University School of Medicine, from January 2014 to December 2024. Data on demographics, preoperative characteristics, surgical techniques, and postoperative outcomes were collected and analyzed descriptively. Twenty-two patients were included, with a median age of 29.5 years (IQR: 24.75–33.75), and 81.8% were male. A laparoscopic-assisted approach was feasible in 86.4% of patients, and the Heineke-Mikulicz technique was used in 81.8%. Postoperative complications occurred in 18.2% (4/22), including transient gastroparesis (13.6%, n = 3) and one repair-site leak (4.5%, n = 1). During a median follow-up of 2.8 years, no reoperations for duodenal stenosis were required, and endoscopic evidence of recurrent duodenal lesions was observed in 2 of 21 evaluable patients. In this retrospective single-center case series, strictureplasty was associated with acceptable postoperative morbidity and satisfactory mid-term outcomes in selected patients with primary proximal duodenal stricturing CD. It may represent a feasible bowel-sparing option when carefully applied, although larger prospective studies are warranted.

## Introduction

Crohn’s disease (CD) can involve any segment of the gastrointestinal tract from the mouth to the anus, with the terminal ileum, ileocecal, or colonic regions most commonly affected [1]. Duodenal involvement is relatively uncommon, occurring in 0.5–4% of cases, and is often asymptomatic [2]. However, primary duodenal CD is increasingly recognized due to heightened emphasis on upper gastrointestinal evaluation during diagnosis [3]. When symptomatic, isolated duodenal CD typically manifests as fibrotic strictures, leading to gastric outlet or duodenal obstruction [4]. Although medical therapies such as proton pump inhibitors and biologics can manage inflammation, fixed fibrotic strictures frequently necessitate mechanical interventions. Surgery is often required for symptomatic duodenal obstruction refractory to medical or endoscopic treatment [4].

Epidemiological trends further highlight the evolving global burden of inflammatory bowel disease (IBD), including CD, underscoring the importance of region-specific insights. Worldwide, CD has transitioned through distinct stages: emergence in developing regions, acceleration in newly industrialized countries, and compounding prevalence in Western societies, driven by environmental and genetic factors [5, 6]. In Asia, incidence rates are rising rapidly, with some newly industrialized regions such as Taiwan reporting annual percentage increases of more than 4% in inflammatory bowel disease incidence, reflecting westernization of lifestyles [6]. Specifically in urban China, the 2016 incidence of IBD reached 10.04 per 100,000 person-years, with CD peaking in young adults (30–34 years) and showing a male predominance, signaling an accelerating burden amid industrialization [7]. Phenotypic differences between Eastern and Western populations are notable: Asian patients often present with distinct clinical features, such as higher rates of perianal involvement and varying treatment responses, potentially linked to environmental triggers and genetic susceptibility [8]. These disparities emphasize the need for region-specific research to address the growing IBD epidemic in Asia [5–7].

Management of proximal duodenal CD poses significant challenges due to its anatomical constraints and high recurrence rates [9]. Traditional surgical approaches include gastrojejunostomy or duodenojejunostomy (bypass), segmental resection, or strictureplasty, with the choice depending on the stricture’s location, extent, number, and concurrent gastrointestinal lesions [10]. No standardized surgical technique exists, requiring a careful balance between symptom relief and complication minimization [3].

Strictureplasty, a bowel-sparing procedure that widens strictures without resection, is a cornerstone in CD surgery, especially for small bowel involvement [11]. Duodenal applications include Heineke-Mikulicz (HM), Finney, and Jaboulay techniques, selected based on stricture length and site [3, 4, 12]. The HM technique is generally used for short strictures, whereas Finney and Jaboulay procedures are considered for longer lesions [13]. Although strictureplasty is well established for small-bowel disease, its application in the duodenum—with its unique anatomy and physiology—remains under investigation and debate [12, 14]. This study aimed to evaluate the safety and mid-term outcomes of strictureplasty for primary proximal duodenal Crohn’s disease at our institution and to provide additional evidence for this uncommon but challenging clinical scenario.

## Materials and methods

In this retrospective single-center case series, we reviewed the electronic medical records of patients with primary duodenal CD who underwent strictureplasty at Sir Run Run Shaw Hospital, Zhejiang University School of Medicine, from January 2014 to December 2024.

Patients were included if they met the following criteria: (1) aged 16–65 years with an established diagnosis of Crohn’s disease based on clinical, endoscopic, radiologic, and histopathologic assessment; (2) a primary proximal duodenal lesion involving the first and second portions of the duodenum, confirmed by endoscopy, computed tomography (CT), gastrointestinal contrast studies, and intraoperative findings; and (3) strictureplasty performed by a consistent surgical team. Exclusion criteria were incomplete medical records or lack of available clinical follow-up beyond 1 year. Collected data included demographics, disease duration, smoking status, medication history, disease behavior (according to the Montreal classification [15]), sites of involvement, surgical details, and postoperative outcomes.

The study adhered to the Declaration of Helsinki and was approved by the Sir Run Run Shaw Hospital Ethics Committee (Approval No. 2023–630-01). Informed consent was waived owing to the retrospective design.

Comprehensive preoperative assessment included upper gastrointestinal endoscopy and contrast imaging to define stricture location, length, and characteristics. Nutritional status was optimized through enteral support. A nasojejunal feeding tube was placed endoscopically to facilitate preoperative nutritional support and to serve as an intraoperative marker for identifying the enterotomy site. In cases with marked gastric wall edema, nasogastric decompression and hypertonic saline lavage were used to reduce inflammation and improve surgical conditions.

In planned laparoscopic-assisted cases, the procedure began with laparoscopic Kocher mobilization of the pylorus, duodenal bulb, and descending duodenum to improve exposure and reduce suture tension. An upper midline incision was then made with wound protection. Conversion to open surgery was performed when laparoscopic mobilization proved technically difficult. Direct open surgery was reserved for patients with post-dilation perforation.

**Fig. 1:**
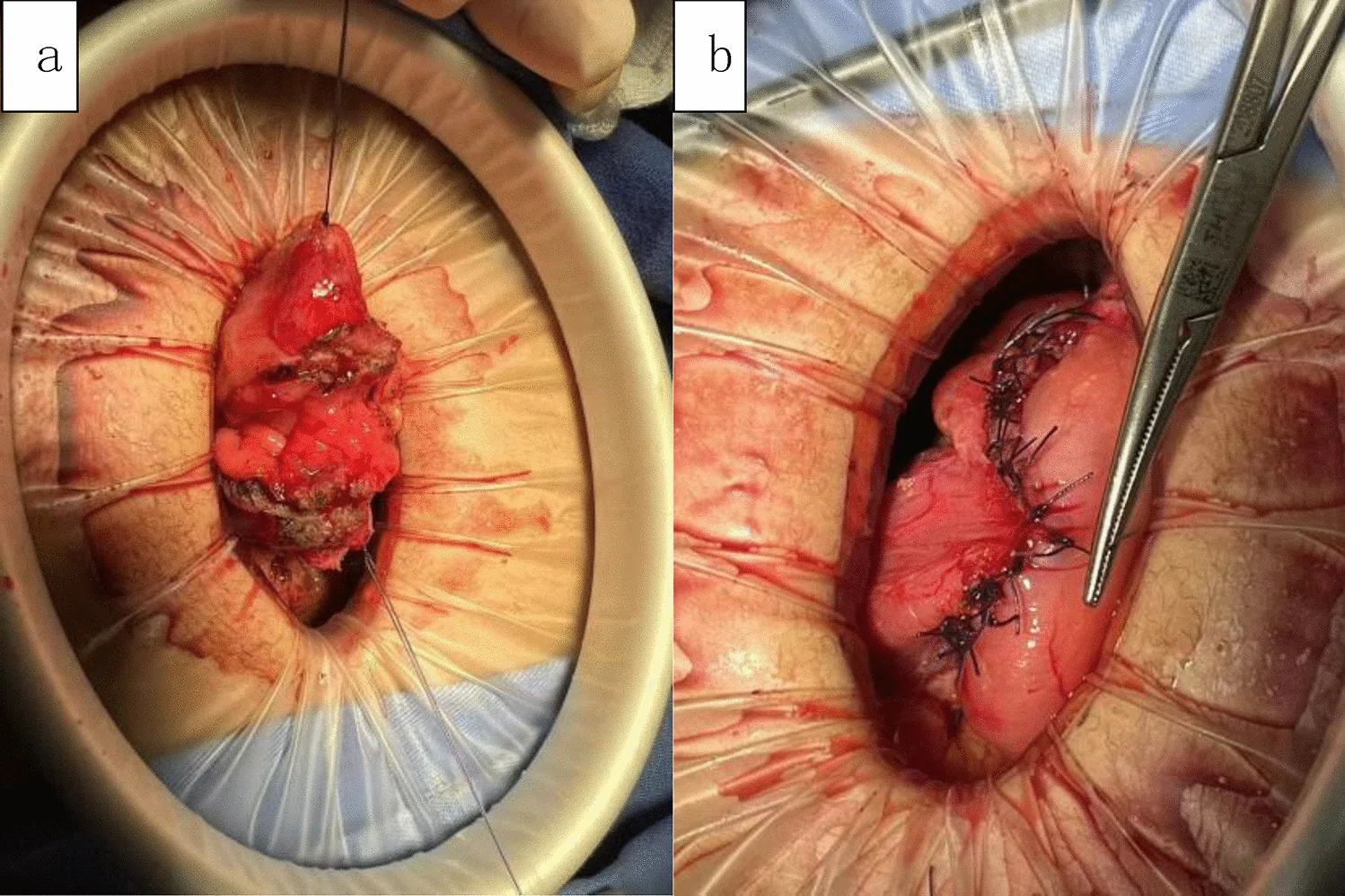
Intraoperative images of Heineke-Mikulicz strictureplasty for a duodenal Crohn’s stricture. **a** Longitudinal incision of the intestinal wall along the antimesenteric border of the duodenum.** b** Transverse closure of the incision using interrupted 3–0 absorbable sutures

The choice of strictureplasty technique was determined by stricture location and length. In this series, the Heineke-Mikulicz (HM) and Finney techniques were used. HM strictureplasty was used for strictures < 5 cm and involved a 4–6 cm longitudinal incision centered on the stricture, followed by transverse closure (Fig. 1a and 1b). Finney strictureplasty was applied to strictures 5–10 cm in length and involved a 10–12 cm longitudinal incision, with the bowel folded into a U-shape for side-to-side suturing. Closure was completed using interrupted 3–0 absorbable sutures. A nasogastric tube was routinely placed intraoperatively for decompression.

**Fig. 2:**
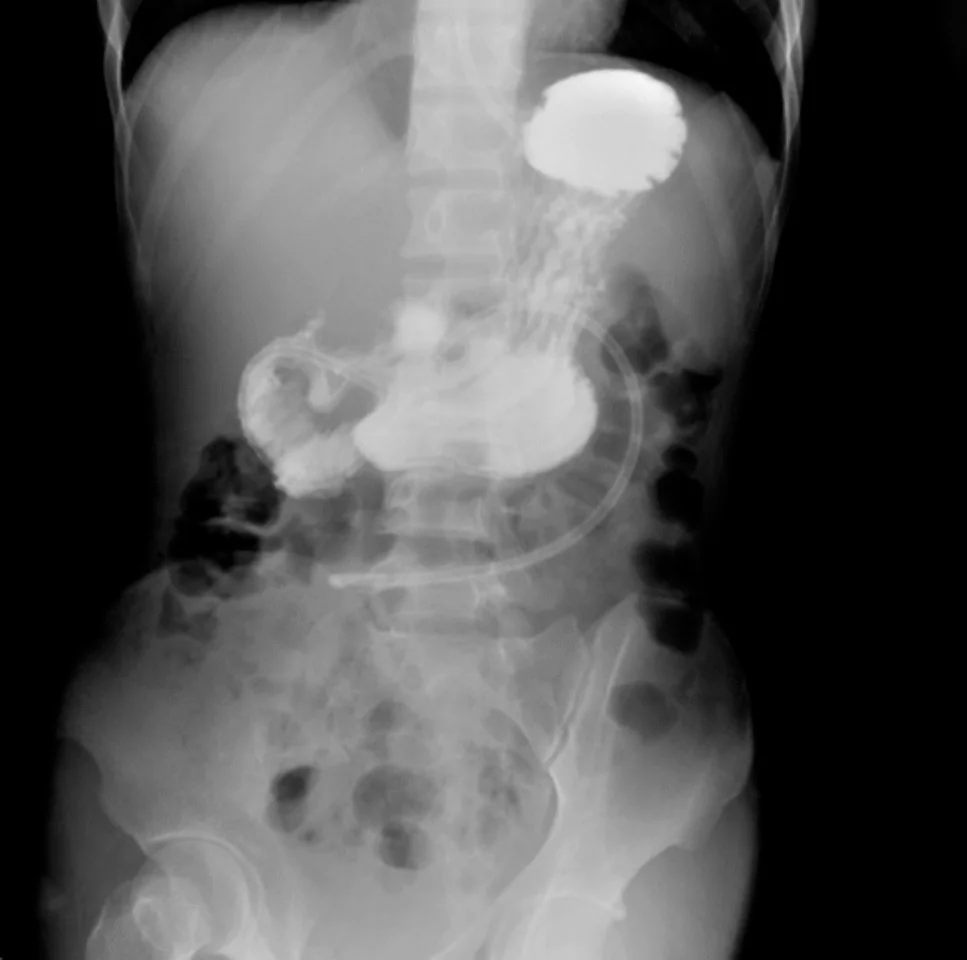
Postoperative upper gastrointestinal contrast study showing improved gastroduodenal passage

Postoperative care emphasized early recovery and close monitoring for complications. Enteral nutrition through the nasojejunal tube was initiated on postoperative day 2. On postoperative day 5, upper gastrointestinal contrast imaging was performed to assess repair-site integrity and gastroduodenal passage; if no leakage or gastroparesis was evident (Fig. 2), the tube was removed and oral intake was resumed gradually. Strategies to prevent postoperative disease recurrence were individualized according to concurrent lesions and patient risk profiles. Follow-up was conducted through outpatient visits, telephone interviews, and periodic assessments for endoscopic or surgical recurrence. Endoscopic recurrence was defined descriptively as recurrent duodenal narrowing and/or inflammatory lesions identified on follow-up upper endoscopy.

**Fig. 3:**
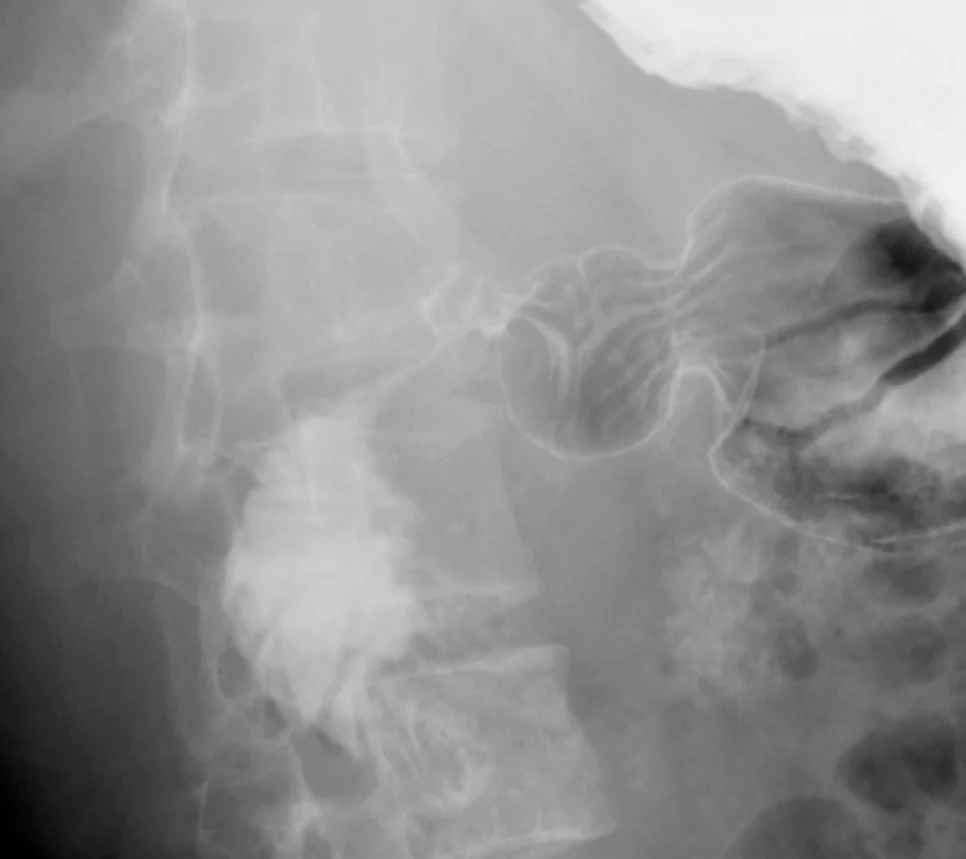
Upper gastrointestinal contrast study showing marked duodenal narrowing and deformity of the duodenal bulb

Data analysis was performed using SPSS version 26.0 (IBM Corp., Armonk, NY, USA). Cases with missing data were excluded from relevant analyses. Continuous variables were reported as mean ± standard deviation (SD) for normally distributed data or median (interquartile range [IQR]) for non-normally distributed data. Categorical variables were expressed as frequencies and percentages. No inferential statistics were performed because of the descriptive nature of the study.

## Results

Between 2014 and 2024, 22 patients underwent strictureplasty for primary duodenal CD. The cohort was predominantly male (81.8%, n = 18), with a median age at surgery of 29.5 years (IQR: 24.75–33.75). Preoperative nutritional status indicated mild malnutrition, with a median body mass index (BMI) of 17.05 kg/m^2^ (IQR: 15.73–19.45) and a median serum albumin level of 40.2 g/L (IQR: 34.25–44.10). The median disease duration was 44 months (IQR: 13.75–77.25), and 27.3% of patients (n = 6) had a history of prior CD-related abdominal surgery (Table 1).

**Table 1:** Baseline characteristics of the study population

Characteristics	Total (n = 22)
Age at surgery*, years	29.5 (24.75–33.75)
Men, n (%)	18 (81.8%)
Disease duration*, months	44 (13.75–77.25)
BMI*, *kg/m*^*2*^	17.05 (15.73–19.45)
Prior CD abdominal surgery, n (%)	6 (27.3%)
Current smoking, n (%)	2 (9.1%)
Preoperative hemoglobin*, g/dL	12.9 (9.50–14.25)
Preoperative albumin*, g/L	40.2 (34.25–44.10)
Preoperative CRP*, mg/L	1.65 (0.48–3.47)
*Preoperative treatment, n (%)*	
5-ASA	1 (4.5%)
Immunomodulators	4 (18.2%)
Biologic therapy	13 (59.1%)
Others	4 (18.2%)
*Endoscopic dilation history, n (%)*	
None	4 (18.2%)
Once	16 (72.7%)
More than once	2 (9.1%)
*Lesion site (Montreal L), n (%)*	
L4 (duodenum only)	5 (22.7%)
L4 + L1 (ileal)	7 (31.8%)
L4 + L2 (colonic)	1 (4.5%)
L4 + L3 (ileocolonic)	9 (40.9%)
*Duodenal stenosis site, n (%)*	
Duodenal bulb / pylorus	11 (50%)
Second portion	11 (50%)
Perianal disease, n (%)	13 (59.1%)

Values in parentheses are percentages unless indicated otherwise; *values are median (interquartile range); 5-ASA, 5-aminosalicylic acid; CRP, C-reactive protein

All patients had proximal duodenal involvement involving the first and second portions of the duodenum. Concurrent disease locations included isolated duodenal disease (L4, 22.7%, n = 5), ileal disease (L4 + L1, 31.8%, n = 7), colonic disease (L4 + L2, 4.5%, n = 1), and ileocolonic disease (L4 + L3, 40.9%, n = 9). Strictures were evenly distributed between the duodenal bulb/pylorus (50%, n = 11) and the second portion of the duodenum (50%, n = 11). Preoperative upper gastrointestinal contrast imaging typically demonstrated marked narrowing and deformity of the duodenal bulb (Fig. 3). Preoperative medical therapy included biologics in 59.1% of patients (n = 13), immunomodulators in 18.2% (n = 4), 5-aminosalicylic acid in 4.5% (n = 1), and other treatments in 18.2% (n = 4). Endoscopic dilation had been attempted in 81.8% of patients (n = 18), most commonly as a single session (72.7%, n = 16). Perianal disease was present in 59.1% (n = 13). Systemic inflammation was mild overall (median C-reactive protein [CRP], 1.65 mg/L; IQR: 0.48–3.47), although nutritional deficiencies included anemia (median hemoglobin, 12.9 g/dL; IQR: 9.50–14.25). Smoking was uncommon (9.1%, n = 2). All patients were referred from the gastroenterology department after prior interventions such as mesalamine, azathioprine, corticosteroids, anti-TNF agents, nutritional support, or anti-inflammatory treatment.

As detailed in Table 2, strictureplasty was successfully performed in all 22 patients. A laparoscopic-assisted approach was used in 86.4% (n = 19), whereas open surgery was required in 13.6% (n = 3), including one conversion because of adhesions and two cases with dilation-related perforation. The HM technique was the most commonly used (81.8%, n = 18) and was generally applied to short strictures, whereas Finney strictureplasty was used in 18.2% (n = 4) for longer lesions. Concurrent procedures were performed in 13.6% (n = 3), including ileal resection (n = 1) and colonic resection (n = 2). Median operative time was 147.5 min (IQR: 122.5–170), and intraoperative blood loss was minimal (median, 20 mL; IQR: 17.5–30).

**Table 2:** Operative details and outcomes

Parameter	N = 22
*Surgical approach*	
Laparoscopic-assisted	19 (86.4%)
Open	3 (13.6%)
*Strictureplasty technique*	
Heineke-Mikulicz (HM)	18 (81.8%)
Finney	4 (18.2%)
*Concurrent procedures*	
None	19 (86.4%)
Ileal resection	1 (4.5%)
Colonic resection	2 (9.1%)
Operative time*, min	147.5 (122.5–170)
Blood loss*, mL	20 (17.5–30)
Postoperative hospital stay*, days	10 (7–12.25)
*Postoperative complications*	
None	18 (81.8%)
Gastroparesis	3 (13.6%)
Repair-site leak	1 (4.5%)
*Postoperative medication*	
None	3 (13.6%)
Azathioprine	2 (9.1%)
Biologics	17 (77.3%)
Endoscopic recurrence	2/21^#^ (9.5%)

Values in parentheses are percentages unless indicated otherwise; *values are median (interquartile range). #Among 21 patients who underwent postoperative endoscopic evaluation; recurrence was defined as recurrent duodenal narrowing and/or inflammatory lesions on follow-up endoscopy

Postoperative complications occurred in 18.2% of patients (n = 4): transient gastroparesis in 13.6% (n = 3), which resolved with conservative management, and one repair-site leak (4.5%, n = 1) that healed after double-tube flushing. The median hospital stay was 10 days (IQR: 7–12.25). Postoperative medication included biologics in 77.3% (n = 17), azathioprine in 9.1% (n = 2), and no maintenance medication in 13.6% (n = 3). At a median follow-up of 2.8 years (IQR: 1.5–4.2), clinical follow-up was available for all 22 patients; however, one patient did not undergo postoperative endoscopic evaluation. Among the 21 patients who underwent postoperative endoscopic evaluation, endoscopic recurrence was observed in 2 (9.5%).

## Discussion

Our study suggests that strictureplasty is a feasible bowel-sparing surgical option for selected patients with primary proximal duodenal Crohn’s disease. In this cohort, the procedure was associated with no reoperation for duodenal stenosis during a median follow-up of 2.8 years, and endoscopic recurrence was observed in 2 of 21 evaluable patients. These findings are clinically relevant because the study population did not represent a low-risk group; rather, many patients had long-standing disease, prior biologic exposure, and previous endoscopic balloon dilation, indicating a treatment-refractory course. In this context, preservation of duodenal continuity without resection or bypass may offer a meaningful clinical advantage.

The postoperative morbidity observed in our series should be interpreted cautiously. Although the overall complication rate was 18.2%, this estimate was derived from a small sample and was driven mainly by transient gastroparesis rather than by multiple major technical failures. Delayed gastric emptying after surgery in the proximal duodenum is biologically plausible and may be related to temporary postoperative edema or motility disturbance in the pyloroduodenal region. In our cohort, these events resolved with conservative management, suggesting that the postoperative risk profile was acceptable and generally manageable. The single repair-site leak should also be interpreted in the context of Crohn’s disease, in which chronic transmural inflammation and fibrosis may compromise tissue quality and healing, even after technically appropriate reconstruction [11, 12].

Our findings should be considered alongside the heterogeneous literature on duodenal Crohn’s disease. Worsey et al. reported symptomatic improvement in all 13 patients treated with duodenal strictureplasty, although 15% required reoperation during follow-up [16]. In contrast, Yamamoto et al. reported reoperation in 69% of 13 patients after duodenal strictureplasty, mainly because of dehiscence, prolonged obstruction, or restricture [12]. Such variation likely reflects differences in patient selection, disease duration, stricture length and location, and the technical difficulty of identifying bowel suitable for reconstruction in a chronically inflamed duodenum. The favorable outcomes in our cohort may therefore relate not only to the procedure itself but also to careful selection of patients with anatomically suitable, predominantly short-segment strictures, most of whom underwent Heineke-Mikulicz strictureplasty.

The absence of reoperation for duodenal stenosis in our series is noteworthy because surgery was generally undertaken after failure of less invasive strategies rather than as an early intervention. More than half of the patients had received biologic therapy preoperatively, and most had undergone prior endoscopic balloon dilation, supporting the interpretation that this was a refractory subgroup. In such patients, strictureplasty may be particularly valuable because it directly addresses fixed fibrostenotic narrowing while preserving bowel length, an important consideration in Crohn’s disease, in which cumulative bowel loss may have substantial long-term consequences. Our findings are therefore consistent with the view that strictureplasty is most applicable when symptoms are driven predominantly by localized fibrotic obstruction rather than by extensive penetrating or diffusely active disease [3, 17].

Another notable aspect of this study is the high rate of minimally invasive surgery. A laparoscopic-assisted approach was feasible in 86.4% of cases and was associated with limited blood loss and an acceptable postoperative hospital stay. Although surgery in the proximal duodenum remains technically demanding because of its retroperitoneal location and its close relationship to the pancreas and biliary tract, increasing experience in advanced minimally invasive inflammatory bowel disease surgery may have expanded the applicability of laparoscopy in selected patients [4, 18]. This trend is consistent with contemporary Crohn’s disease surgery in the biologics era, in which minimally invasive approaches are increasingly used in appropriately selected patients [19]. This may be particularly relevant in relatively young patients with chronic disease burden and limited nutritional reserve, as reflected by the low median BMI in our cohort.

These findings should also be interpreted in relation to alternative treatment strategies. Medical therapy remains the cornerstone of management in duodenal Crohn’s disease, but established fibrotic strictures often respond incompletely once obstructive symptoms develop [17]. Endoscopic balloon dilation may be effective in short and accessible strictures, although repeated interventions are frequently required and technical limitations are common in the duodenum [17, 18]. Bypass procedures continue to play an important role in selected complex cases. Notably, a recent meta-analysis suggested that bypass may be associated with lower treatment failure than stricturoplasty in duodenal Crohn’s disease, underscoring the importance of careful patient selection when a bowel-sparing approach is considered [14]. Resection is sometimes unavoidable, but it is generally more invasive and technically demanding in the proximal duodenum [4]. Within this therapeutic spectrum, strictureplasty appears to represent a reasonable bowel-preserving option for appropriately selected patients.

Several limitations should be acknowledged. This was a retrospective single-center case series with a limited sample size, which is understandable given the rarity of primary proximal duodenal Crohn’s disease but nonetheless restricts statistical power and generalizability. The absence of a control group precludes formal comparison with bypass, resection, or repeated endoscopic treatment. In addition, the outcomes may partly reflect the experience of a specialized surgical team and therefore may not be directly reproducible across all practice settings. Finally, although the median follow-up of 2.8 years provides meaningful mid-term information, longer surveillance is needed to better define the durability of this approach in a chronic relapsing disease. Despite these limitations, our data suggest that strictureplasty may be a valid bowel-sparing option for selected patients with primary proximal duodenal Crohn’s disease, with preservation of luminal continuity and an acceptable postoperative risk profile.

## Conclusions

In this retrospective single-center case series, strictureplasty appeared to be a feasible bowel-sparing surgical option for selected patients with primary proximal duodenal stricturing Crohn’s disease. The procedure was associated with satisfactory mid-term outcomes, preservation of duodenal continuity, and an acceptable postoperative risk profile. Given the retrospective design, limited sample size, and lack of a control group, these findings should be interpreted cautiously. Larger prospective multicenter studies are needed to confirm these results and to clarify the role of strictureplasty relative to bypass, resection, and endoscopic treatment strategies.

## Data Availability

The data that support the findings of this study are available from the corresponding author upon reasonable request.
